# A comparative study of the phenolic compounds and antioxidant potential of *Morchella elata Fr.* and *Morchella esculenta (L).Pers*

**DOI:** 10.1038/s41598-026-42929-7

**Published:** 2026-04-03

**Authors:** Ali Murat Kesemen, Mustafa Aybar, Erol Tunca, Zeynep Berin Celebi, Yusuf Uzun, Sevgi Kolaylı

**Affiliations:** 1 Department of Hotel, Restaurant and Catering Services, Artvin Vocational School, Artvin Çoruh University, Artvin, Turkey; 2 Department of Forest Engineering, Faculty of Forestry, Karabük University, Karabük, Turkey; 3 Faculty of Science, Department of Chemistry, Karadeniz Technical University, Trabzon, Turkey; 4 Department of Biochemistry, Faculty of Pharmacy, Karadeniz Technical University, Trabzon, Turkey; 5 Department of Biochemistry (Pharmacy), Graduate School of Health Sciences, Karadeniz Technical University, Trabzon, Turkey; 6 Department of Pharmaceutical Botany, Faculty of Pharmacy, Van Yüzüncü Yıl University, Van, Turkey; 7 Nakhchivan State University, Nakhchivan, Azerbaijan

**Keywords:** Mushroom, morel, M. elata, M. esculenta, antioxidant, phenolic substance, Biochemistry, Biotechnology, Chemical biology, Chemistry, Plant sciences, Zoology

## Abstract

Wild, edible lamb’s ear mushrooms (*Morchella* spp.) are highly nutritious and have a distinctive aroma, making them popular worldwide. This study compared the nutritional composition and bioactive constituents of *Morchella elata* Fr. and *Morchella esculenta* (L.) Pers. collected from the province of Artvin in the Eastern Black Sea region of Türkiye. The dry matter content was approximately 18% in both species. Color analysis indicated that *M. elata* exhibited significantly lower Hunter L values (16.00), reflecting a darker appearance compared to *M. esculenta* (41.66 ± 0.31). Crude protein content was 24.85 ± 0.50 g/100 g DW in *M. elata* and 31.14 ± 0.50 g/100 g DW in *M. esculenta*. The total phenolic compound content was significantly higher in *M. elata* (15.76 ± 0.56 mg GAE/g DW) than in *M. esculenta* (7.41 ± 0.17 mg GAE/g DW). The antioxidant capacity of the methanolic extracts was measured using the FRAP and DPPH methods, and *M. elata* emerged as superior in that regard. HPLC-PDA analysis results revealed that *M. elata* is rich in protocatechuic acid and *t*-cinnamic acid, while *M. esculenta* has higher epicatechin, pinocembrin, and chrysin contents. The findings suggest that the dark-colored *M. elata* species may be more valuable than *M. esculenta* in terms of antioxidant capacity.

**Supplementary Information:**

The online version contains supplementary material available at 10.1038/s41598-026-42929-7.

## Introduction

Edible mushrooms are important natural resources in terms of both nutrition and health due to their high dietary value and functional properties. In addition to being rich in protein, vitamins, minerals, and dietary fiber, they also contribute to healthy nutrition with their low-fat content^1,2^.Furthermore, these organisms are rich in phenolic compounds, polysaccharides, and other bioactive components, exhibiting strong antioxidant, antimicrobial, and immune-modulating properties. Edible mushrooms are therefore widely used not only in the food industry, but also in pharmaceutical and functional food research^3^.

The genus *Morchella* (morels), belonging to the phylum *Ascomycota*, is one of the most valuable groups of edible and medicinal mushrooms due to its distinctive honeycomb-like cap structure, unique aroma, and nutritional benefits. Found mainly in temperate forest ecosystems of the Northern Hemisphere, *Morchella* species are typically collected from natural habitats during the spring and are difficult to cultivate, which enhances their commercial value^4,5^. These mushrooms are rich in proteins, fibers, B vitamins, vitamin D, and essential minerals such as potassium, phosphorus, and iron. In addition to their culinary appeal, they contain phenolic compounds and antioxidants that contribute to their therapeutic potential and traditional use in treating ailments such as fatigue, muscle pain, and respiratory conditions^6,7^. Species such as *Morchella esculenta*, *Morchella elata*, and *Morchella vulgaris* are especially prized for both gastronomic and pharmacological purposes, making the genus *Morchella* an economically significant and scientifically important group traded and studied worldwide^8,9^.

Türkiye is characterized by a rich diversity of mushrooms, the Eastern Black Sea region being particularly notable for its high fungal biodiversity. Studies in this area have shown that agaricoid, boletoid, and *Morchella* species are widely distributed. Members of the genus *Morchella* are primarily found in temperate regions of the Northern Hemisphere, including Europe, North America, Asia, and the Mediterranean^9,10^.These fungi occur naturally in forested habitats with moist, well-drained, and organic matter-rich soils, and are especially abundant during the spring months under pine, oak, poplar, and other deciduous trees. *M. esculenta* and *M. elata* exhibit both mycorrhizal and saprophytic lifestyles, enabling them to form symbiotic associations with tree roots while contributing to the decomposition of organic matter. Their ecological adaptability and widespread distribution highlight their significant role in temperate forest ecosystems^11,12^.

Artvin province has an ecosystem ideally suited to the growth of wild edible mushrooms due to its rich vegetation, high altitude, and year-round rainy climate. The area’s cool and humid air conditions, especially in spring and autumn, support the natural growth of valuable species such as *Morchella*,* Cantharellus*, and *Lactarius*. The prevalence of forested areas and microclimatic diversity enhance mushroom species’ richness while also contributing to an extended production season. These characteristics make Artvin one of Türkiye’s important mushroom-producing regions^13,14^.

Although numerous studies have investigated cultivated and wild edible mushrooms in the Northern Hemisphere, only limited information is available concerning naturally occurring edible wild mushrooms and species with high antioxidant capacity in Türkiye. This study aimed to comparatively investigate the nutritional components, bioactive contents, and antioxidant potential of two economically important mushroom species, *Morchella elata Fr.* and *Morchella esculenta (L.) Pers.*, both naturally occurring in the Eastern Black Sea region (Artvin) of Türkiye. The study arose from the need to expand the limited scientific knowledge currently available regarding the bioactive properties of wild edible mushrooms in the region.

## Materials and methods

### Sample collection and characterization

**Fig. 1:**
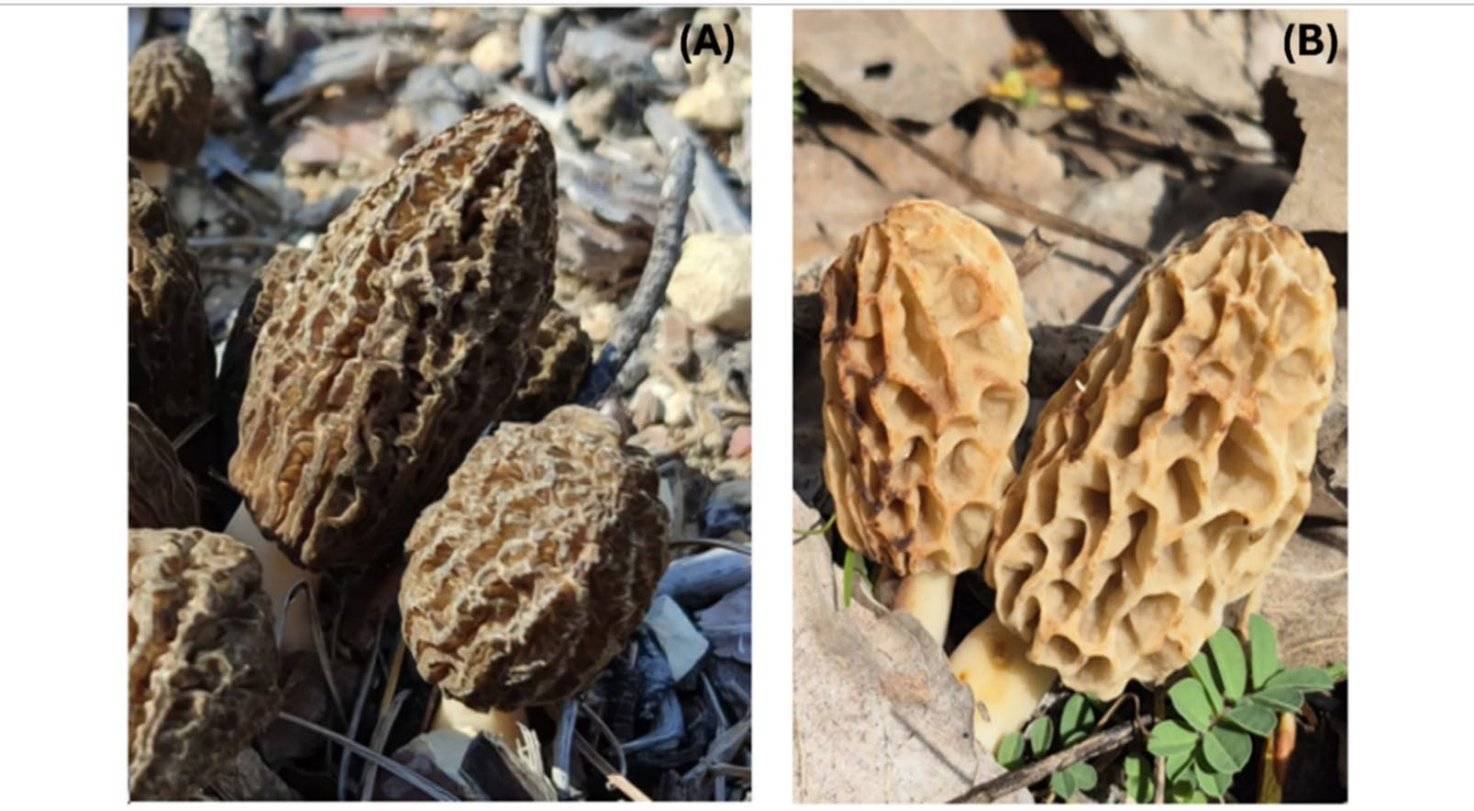
(**A**) *Morchella elata* Fr.; (**B**) *Morchella esculenta* (L.) Pers. The photographs were taken by Dr. Ali Murat Kesemen.

The morel samples collected during fieldwork were transported to the laboratory in clean polyethylene bags at + 4 °C (Fig. 1). The samples were divided into two subgroups for species identification and chemical analysis. The part set aside for analysis was dried in an oven at 40 °C and then ground into powder in a grinder. Details of the collection sites, including their geographical locations and coordinates, are provided in Table 1. The two *Morchella* species studied from the Artvin region, *Morchella esculenta* (L.) Pers. (yellow morel) and *Morchella elata* Fr. (black morel), were identified and classified based on their distinctive macroscopic and microscopic features and were formally named by Prof. Dr. Yusuf Uzun (Van 100. Yıl University, Department of Pharmaceutical and Botany, Turkiye). Both species emerge in spring on moist, well-drained, and organic matter-rich soils, frequently colonizing post-fire habitats, and display dual mycorrhizal and saprophytic lifestyles. Macroscopically, *M. esculenta* is characterized by a light-colored, yellow to golden-brown cap with oval to rounded alveoli and a generally broad morphology, whereas *M. elata* exhibits a darker, conical, and elongated cap with vertically arranged and deeper pits. Both species possess a hollow, brittle, and sponge-like internal structure. Microscopically, the asci of both species are eight-spored; *M. esculenta* produces smaller, hyaline, oil-droplet-containing ascospores measuring 17–23 × 9–13 μm, while *M. elata* has larger spores (20–28 × 12–16 μm) sometimes associated with pigmented paraphyses^7,15^. The excipulum cells of both species are hyaline to light brown and range from globose to angular in shape. These morphological and microscopic characteristics were crucial for accurate species identification and reflect the ecological adaptations of each taxon^16,17^.

**Table 1:** The species, areas, and coordinates of the mushroom samples.

Species	Region	Coordinates	Altitude (m)	Season	Fungary om nu
*M. esculenta*	Şavşat-Tepeköy/Artvin	41°17’58.4” 42°13’35.7”	750	20 April, 2025	ME-01
*M. elata*	Yusufeli-Demirkent/Artvin	40°53’10.4” 41°44’28.5”	790	21 April,2025	ME-02

### Extraction of samples

Methanolic extracts were prepared from dried samples for antioxidant properties and phenolic compound analyses. Briefly, 3 g of dry mushroom powder was transferred to Falcon tubes containing 30 mL of 70% methanol solution. The tubes were kept in an ultrasonic bath for 2 h, and then stirred in a shaker for 24 h. The resulting extracts were filtered first through coarse- followed by fine-pored (Whatman No. 1) filter paper. The filtrates were divided into aliquots and stored in a deep freezer until analysis^18^. Three samples were used in each study, and arithmetic means and standard deviations are given as the average of the three samples.

### Determination of physicochemical parameters

Moisture content in the mushroom samples was assessed following the official AOAC procedure^19^. Approximately 5 gram of freshly collected sample were weighed and dried in a hot air oven set at 105 ± 2 °C until a consistent weight was reached. The moisture level was then calculated based on the weight difference before and after drying. Crude protein content was determined using the Kjeldahl method, the nitrogen content being multiplied by a conversion factor of 6.25.

The color characteristics of the mushroom samples were measured using the Hunter Lab color scale^20^. In this system, the L* value reflects the lightness of the sample, where 0 represents black and 100 corresponds to white. The a* value indicates color variation along the red-green axis, with positive values showing a shift toward red, while negative values indicate green tones. The b value represents the yellow-blue spectrum, with positive readings suggesting yellowness and negative ones indicating blueness.

## Determination of total phenolic content (TPC)

The total phenolic content (TPC) was measured spectrophotometrically following the method described by Slinkard and Singleton^21^, with slight modifications. Briefly, 20 µL of each sample was mixed with 400 µL of diluted Folin–Ciocalteu reagent (0.25 N), followed by the addition of 400 µL of a 7.5% sodium carbonate solution and 750 µL of distilled water. The mixtures were incubated under appropriate conditions to allow color development. After incubation, absorbance was recorded at 765 nm. The results were expressed as mg of gallic acid equivalents (mg GAE/100 g dry weight, DW), calculated based on the standard calibration curve (Supplement S1).

### Determination of total flavonoid content (TFC)

The total flavonoid content (TFC) was measured using a colorimetric method with a minor modification, in which aluminum chloride was replaced by aluminum nitrate^22^. In this assay, 250 µL of the methanolic extract was mixed with 50 µL of 10% aluminum nitrate Al(NO₃)₃ and 50 µL of 1.0 M ammonium acetate (NH₄CH₃COO). The mixture was then diluted with 99.9% methanol to a final volume of 2.6 mL. After incubation at 25 °C for 40 min, absorbance was measured at 415 nm using a UV-Vis spectrophotometer. A standard calibration curve was constructed using quercetin solutions prepared in methanol at concentrations ranging from 0.031 to 0.50 mg/mL. All standard dilutions were performed using the same solvent. The results were expressed as mg of quercetin equivalents (mg QE/100 g dry weight, DW), calculated based on the standard calibration curve (Supplement S2).

### Determination of antioxidant potential

The total antioxidant capacity of the methanolic extract was evaluated using the ferric reducing antioxidant power (FRAP) assay, originally described by Benzie and Strain^23^. In this procedure, 1.5 mL of freshly prepared FRAP reagent, consisting of 300 mM acetate buffer (pH 3.6), 10 mM TPTZ (2,4,6-Tris(2-pyridyl)-s-triazine), and 20 mM FeCl₃ in a 10:1:1 ratio, was mixed with 50 µL of the extract. The mixture was incubated at 37 °C for 4 min to allow the reaction to occur. Following incubation, absorbance was recorded at 593 nm using a UV-Vis spectrophotometer. A standard calibration curve was generated using Trolox at concentrations ranging from 31.25 to 1000 µmol/L. The results were expressed as mmol of Trolox equivalents per gram of dry sample weight (DW), calculated based on the standard calibration curve (Supplement S3).

The DPPH free radical scavenging activity of the extract was assessed using a spectrophotometric approach based on the method described by Molyneux^24^. In this assay, 0.75 mL of a 100 µM DPPH solution was combined with 0.75 mL of the extract. The reaction mixture was then incubated in the dark at 25 °C for 50 min to prevent light-induced degradation. After incubation, absorbance was recorded at 517 nm. In order to determine the SC_50_ value, defined as the concentration of the extract required to scavenge 50% of DPPH radicals, six different concentrations of the methanolic extract were prepared and analyzed under identical experimental conditions. The absorbance data were used to generate a dose–response curve, from which the SC_50_ value was calculated. The results were expressed in mg/mL and determined based on the standard calibration curve (Supplement S4).

### Determination of phenolic composition using HPLC-PDA

In this study, a liquid–liquid extraction technique was employed for a more effective identification of the phenolic compounds in methanolic mushroom extract^25^. First, 10 mL of the methanolic extract was concentrated by evaporation at 40 °C using a rotary evaporator (IKA Werke, Staufen, Germany). The dry residue obtained was then dissolved in 10 mL of purified water, the pH being adjusted to 2 using HCl. Three sequential extractions were then carried out using diethyl ether and ethyl acetate as solvent^21^. Following evaporation of the organic solvents using a rotary evaporator, the remaining residue was dissolved in 2 mL of methanol, filtered through a 0.45 μm RC membrane filter, and injected into the HPLC system for analysis.

The phenolic profile of the extract was determined using an HPLC system coupled with a photodiode array detector (Shimadzu LC-20AT, Kyoto, Japan). The analysis was performed on a C18 reverse-phase column (250 mm × 4.6 mm, 5 μm; GL Sciences, 5020 − 01732). Twenty-six phenolic compounds were used to construct the calibration curve. The mobile phase consisted of two solvents: solvent A was a mixture of acetonitrile and water (70:30, v/v), while solvent B was 2% acetic acid in water. Both standards and samples were injected at a volume of 20 µL. The column temperature kept constant at 30 °C, and the flow rate was set at 1.0 mL/min^26^.

### Statistical analysis

Statistical analyses were performed using SPSS Statistics for Windows version 22.0 software (IBM Corp., Armonk, NY, USA). Differences between two groups were evaluated using Student’s t-test, and *p* < 0.05 was considered statistically significant.

## Results and discussion

This study investigated selected chemical and bioactive properties of two distinct *Morchella* species, representing different color morphotypes, collected from the Artvin region of Türkiye (Fig. 1).

Table 2 summarizes the moisture content, color parameters, and crude protein levels of the samples. The dry matter contents of the two *Morchella* species were comparable, averaging approximately 18%. Edible wild mushrooms typically exhibit a high moisture content, which generally ranges between 80% and 90%, depending on the species, environmental factors, and harvest timing. With drying, moisture levels decrease to 10–20%. Moisture content plays a critical role in determining the shelf-life, storage stability, and processability of mushrooms. Water content also significantly influences technological attributes, including nutritional quality and extract yield^27^.

**Table 2:** The colors and crude protein values of the morel samples.

Analysis	M. elata	M. esculenta
Dry matter %	18.49 ± 0.02^a^	18.03 ± 0.03^a^
Crude Protein (g/100 g DW)	24.85 ± 0.50^a^	31.14 ± 0.50^b^
Color (Hunter L*)	16.34 ± 0.72 ^a^	41.66 ± 0.31^b^
Color (Hunter a*)	3.48 ± 0.54 ^a^	15.42 ± 1.67^b^
Color (Hunter b*)	3.99 ± 0.85 ^a^	21.76 ± 0.94^b^

According to the Kjeldahl method, the crude protein content varied between 24% and 31%. The findings revealed that the light-colored *M. esculenta* species contained higher levels of crude protein than the other species. Edible wild mushrooms are considered an important source of protein, with crude protein contents generally ranging from 15% to 40%, depending on the species. Table 3 summarizes the crude protein contents reported in different previous studies for various *Morchella* species. Accordingly, the crude protein content in *M. esculenta* has been reported as 20.64^28^, 39.35%^29^, and 29.00%^30^. Studies of *M. conica* have reported that crude protein contents vary between 15% and 32%^30^. In the present study, the crude protein content in the *M. esculenta* species was quite high, at 31%. However, since we found no other study of the crude protein content of the *M. elata* species in the current literature, no comparative evaluation was possible. These differences may arise depending on factors such as the analysis methods used, the region where the samples were collected, and environmental conditions^27^.

The color parameters of both *Morchella* species used in this study were analyzed using the Hunter Lab system. The Hunter L value represents color lightness and darkness, with a low L value indicating a darker color. According to the measurement results, the Hunter L value of the species *M. elate* was 16.34 ± 0.72, indicating that this species is darker than *M. esculenta*. The Hunter a* value expresses the redness and greenness of natural samples, with higher values representing redness and lower values representing greenness.

**Table 3:** Total phenolics and antioxidant properties of various *Morchella* species.

Morchella species	Total Phenolic content (µg GAE/mg DW)	Total Flavanoid content (µg QE/mg DW)	Total Protein (g/100 g)	Refs.
*M. conica*	25.38 ± 0.70	0.24 ± 0.01	–	^4^
*M. esculenta*	21.33 ± 1.40	0.25 ± 0.03	–	^4^
*M. crassipes*	18.59 ± 0.70	0.47 ± 0.05	–	^4^
*M. rotunda*	16.98 ± 1.03	0.59 ± 0.01	–	^4^
*M. angusticeps*	16.55 ± 0.98	0.26 ± 0.04	–	^4^
*M. elata*	15.36 ± 0.05	0.30 ± 0.01	–	^4^
*M. deliciosa*	12.36 ± 1.21	0.15 ± 0.02	–	^4^
*M. esculenta*	–	–	20.64 ± 0.06	^28^
*M. conica var. costata*	–	–	30.78	^31^
*M. esculenta*	–	–	39.35 ± 0.35	29
*M. esculenta*	–	–	29.70	^30,32^
*M. conica*	–	–	15.2–32.3	^33^
*M. esculenta*	–	–	26.8	^33^
*M. esculenta*	–	–	32.7	^34–36^
*M. dunalii*	26	–	–	^2^
*M. importuna A*	18	–	–	^2^
*M. importuna B*	19	–	–	^2^
*M. deliciosa*	17	–	–	^2^
*M. mediterraneensis*	20	–	–	^2^
*M. purpurascens*	25	–	–	^2^
*M. esculenta*	18.5–23.8	–	–	^37^
*M. conica*	4.30 ± 0.12 mg/g	0.38 ± 0.00	–	^38^
*M. conica* (Yunnan)	6.407 ± 0.171 mg GAE/g DW	–	–	^39^
*M. conica* (Tibet)	5.116 ± 0.045 mg GAE/g DW			^39^
*M. conica* (Xinjiang)	6.350 ± 0.187 mg GAE/g DW			^39^

According to the results from the present study, *M. elata* has a lower a* value than *M. esculenta*, indicating that the former species has a more greenish color tone. The Hunter b value indicates the distribution of color tone on the yellow-blue axis, positive b* values indicating yellow tones, while negative values indicate blue tones. In this study, *M. elata* species exhibited a lower b* value, indicating that this species has a more bluish color tone. Significant differences in color parameters were thus observed among the *Morchella* spp. subspecies examined. This study reports, for the first time in the literature, the Hunter color values of *M. elata* and *M. esculenta*, revealing significant differences in their color parameters, with *M. elata* exhibiting a darker, greener, and more bluish tone than *M. esculenta*.

The findings for the total phenolic and flavonoid content are presented in Table 4. Analysis of the dried samples revealed a TPC value of 15.76 mg GAE/g in *M. elata*, approximately double the 7.41 mg GAE/g in *M. esculenta*. The findings obtained are similar to those in a previous study of *M. elata*, which reported a TPC value of 15.36 mg GAE/g^4^. In contrast, another study, of *M. esculenta*, reported TPC values from 18.5 to 23.8 mg GAE/g^37^. Similarly, the TFC was also higher in *M. elata*. This result shows that *M. elata* has a richer profile than *M. esculenta* in terms of both phenolic compounds and flavonoid content. The reported TFC was 0.30 mg QE/g in *M. elata* and 0.25 mg QE/g in *M. esculenta*^4^. These data indicate that *M. elata* has a higher TFC than *M. esculenta.*

**Table 4:** The phenolic contents and antioxidant properties of the *Morchella* spp.

Analysis	M. elata	M. esculenta
TPC (mg GAE/g DW)	15.76 ± 0.56^b^	7.41 ± 0.17^a^
TFC (mg QE/g DW)	2.37 ± 0.21^b^	1.45 ± 0.06^a^
Total antioxidant capacity FRAP (mg Trolox/g DW)	4.04 ± 0.14^b^	3.16 ± 0.02^a^
DPPH radical scavenging activity SC_50_ (mg/mL)	0.67 ± 0.01^a^	1.12 ± 0.09^b^

In a different study involving *M. conica*, TPC was reported as 4.30 ± 0.12 mg GAE/g, and TFC content as 0.38 mg QU/g^38^. The values reported in the previous literature were lower than those in the present study. This finding indicates that *Morchella* species collected from the Artvin region are richer in total phenolic and flavonoid content and suggests that these species have significant potential in terms of bioactive compounds.

Polyphenols are multifunctional secondary metabolites produced by plants and fungi. Their levels vary depending on environmental conditions, such as temperature, pollution, stress, and disease, as well as on the botanical characteristics of the organism. TPC is an important distinguishing feature for natural sources, with products containing higher TPC generally exhibiting greater bioactive properties^37,40^. Many epidemiological and clinical studies have demonstrated that dietary intake of foods rich in phenolic compounds is inversely associated with oxidative stress–related cardiovascular morbidity^7,41–43^. In a prospective, 10-year cohort study from Spain, higher consumption of flavonoid-rich foods such as fruits, vegetables, and cocoa was associated with an approximately 1.85-fold decrease in the risk of cardiovascular disease^44^.

The antioxidant capacities of methanol extracts of the morel mushrooms were evaluated using two different methods, FRAP and DPPH assays. The FRAP assay measures total antioxidant capacity, while the DPPH method measures free radical scavenging ability. The results revealed the dark-colored *M. elata* species had a higher FRAP value, indicating a stronger total antioxidant capacity. No significant difference was detected between the two species in terms of total antioxidant capacity. The DPPH radical scavenging activities of the morel extracts were calculated in terms of SC_50_ values, a low value indicating high radical scavenging capacity. The extracts of both species effectively scavenged the DPPH free radical, the *M. elata* species exhibiting a stronger radical scavenging ability than *M. esculenta.* The mushroom species recognized worldwide for their high antioxidant potential include Morel (*Morchella spp*.)^6^, Reishi (*Ganoderma lucidum*)^45^ and Shiitake (*Lentinula edodes*)^46^.

In a previous study, in vitro experiments evaluated three morel species (*M. rufobrunnea*, *M. sextelata*, and *M. americana*) in terms of their antioxidant and anti-inflammatory activities. The results indicated that both aqueous and methanolic extracts of these species exhibited similar chromatographic and biological activity profiles, irrespective of their source (cultured or wild-collected) or phylogenetic position. Notably, the extracts demonstrated potent antioxidant effects, particularly by inhibiting lipid peroxidation, highlighting their significant biological relevance. Furthermore, the extracts exhibited substantial inhibitory activity against cyclooxygenase enzymes (COX-1 and COX-2), underscoring their considerable anti-inflammatory potential^47^.

Significant differences were observed between the examined *Morchella* species in terms of TPC and antioxidant capacity. In particular, the dark-colored *M. elata* exhibited a markedly higher antioxidant capacity. These findings are also consistent with previous reports. One such study revealed a positive correlation between pigmentation density and antioxidant potential in various mushroom species^8^. The elevated phenolic content and corresponding antioxidant activity observed in the darker *Morchella* species parallel similar trends reported for other natural products, such as bee products (honey, pollen, and propolis), as well as fruits and vegetables^20,48^. In these matrices, the high abundance of polyphenolic compounds (including flavonoids, tannins, and anthocyanins) not only contributes to the darker coloration but also enhances their antioxidant and other bioactive properties^48^.

The phenolic components of the methanol extracts of the morel mushrooms were analyzed using the HPLC-PDA method^26^. In this study, 26 distinct phenolic standards were used as references, and the results obtained being presented in Table 5. The HPLC chromatograms of both morel species are presented in Supplements 6 and 7. The analyzed phenolic compounds were categorized into two main classes, phenolic acids and flavonoids, each of which was further subdivided into specific subclasses. Significant differences were observed in the phenolic profiles of the morel extracts. The methanolic extract of *M. elata* was particularly abundant in protocatechuic acid and trans-cinnamic acid, whereas *M. esculenta* contained higher levels of epicatechin, chrysin, and pinocembrin. The concentrations of the remaining phenolic compounds analyzed were below the limit of quantification^26^. A study conducted using a similar methodology to the present research reported that protocatechuic acid was the predominant compound among the 31 phenolic standards analyzed in the methanolic extract of *Morchella steppicola*, followed by vanillic acid, *p*-hydroxybenzoic acid, and gallic acid^49^. Similarly, protocatechuic acid was identified as the major component in *M. elata*, although this compound was below the detection limit in *M. esculenta*. Another study described *M. pulchella* as rich in caffeic acid and protocatechuic acid^5^.47 A study involving *M. sextelata* using UPLC–tandem mass spectrometry identified 44 phenolic compounds, including gallic acid, protocatechuic acid, DL-4-hydroxyphenylactic acid, methyl 2,4-dihydroxyphenylacetate, salicylic acid, 4-hydroxybenzaldehyde, 4-hydroxyacetophenone, and luteolin as the main components^50^. Another study conducted with *M. esculenta* compared samples collected from Portugal and Serbia in terms of various nutrients and bioactive compounds^51^.

**Table 5:** Phenolic compounds of the samples identified by HPLC-PDA.

	Phenolic Standards (µg phenolic/ 100 g DW)	*M. elata*	*M. esculenta*
Phenolic acids	Hydroxybenzoic acids		
	*p-*OH Benzoic acid	< LOD	< LOD
	Protocatechuic acid	3312.30 ± 256.30	< LOD
	Gallic acid	< LOD	< LOD
	Chlorogenic acid	< LOD	< LOD
	Syringic acid	< LOD	< LOD
	Ellagic acid	< LOD	< LOD
	Vanillic acid	< LOD	< LOD
	Hydroxycinnamic acids		
	*t*-cinnamic acid	419.70 ± 149.05	< LOD
	Ferulic acid	< LOD	< LOD
	*p*-Coumaric acid	< LOD	< LOD
	Caffeic acid	< LOD	< LOD
	Caffeic acid phenethyl ester (CAPE)	< LOD	< LOD
Flavonoids	Flavonol		
	Rhamnetin	< LOD	< LOD
	Quercetin	< LOD	< LOD
	Rutin	< LOD	< LOD
	Myricetin	< LOD	< LOD
	Galangin	< LOD	< LOD
	Flavan-3-ols		
	Epicatechin	< LOD	3301.50 ± 431.00
	Catechin hydrate	< LOD	< LOD
	Flavones	< LOD	< LOD
	Chrysin	< LOD	592.90
	Daidzein	< LOD	< LOD
	Apigenin	< LOD	< LOD
	Luteolin	< LOD	< LOD
	Flavanones		
	Pinocembrin	< LOD	866.70 ± 71.66
	Hesperetin	< LOD	< LOD
	Naringenin	< LOD	< LOD
	Stilbenes		
	Resveratrol	< LOD	< LOD

(-): < LOD (limit of detection).

While the total protein content in both samples was approximately 10–12%, significant differences have been reported in mineral matter, fatty acid, and sugar compositions^49^. The same study also reported that TPC differed significantly, with protocatechuic acid, gallic acid, and p-coumaric acid being detected in varying proportions in morel mushrooms. However, compared with the findings of the present study, it may be concluded morel species obtained from the Artvin region are richer in bioactive compounds.

## Conclusion

This study represents the first comparative investigation of the nutritional and bioactive properties of two economically important Morchella species, *M. elata* Fr. and *M. esculenta* (L.) Pers., from Arvin in the Eastern Black Sea region of Türkiye. While *M. esculenta* exhibited higher crude protein content, *M. elata* demonstrated significantly greater TPC and a superior antioxidant capacity, as determined by FRAP and DPPH assays. HPLC-PDA analysis revealed distinct phenolic profiles: *M. elata* was rich in protocatechuic and trans-cinnamic acids, while *M. esculenta* contained higher levels of epicatechin, chrysin, and pinocembrin. Notably, no phenolic compounds were shared between the two species among the 26 standards analyzed.

These findings indicate that the dark-colored *M. elata* may possess greater biological and antioxidant potential than *M. esculenta*. Moreover, the rich vegetation and diverse climate of the Artvin region support the natural growth of Morchella species, emphasizing the importance of preserving these habitats for the sustainability of bioactive-rich morels.

## Supplementary Information

Below is the link to the electronic supplementary material.

Supplementary Material 1

## Data Availability

All data generated or analyzed during this study are included in this published article.
