# Supplementary material for: A comparative study of the phenolic compounds and antioxidant potential of Morchella elata Fr. and Morchella esculenta (L).Pers
Source: Sci Rep. 2026 Apr 3;16:21882. doi: 10.1038/s41598-026-42929-7 (PMC13365187; doi:10.1038/s41598-026-42929-7)
Supplement: Supplementary file 1 — Supplementary Material 1 [file 41598_2026_42929_MOESM1_ESM.docx]

**Supporting Information**

for

A Comparative Study on Phenolic Compounds and Antioxidant Potential of *Morchella elata* Fr. and *Morchella esculenta* (L).Pers

Department of Hotel, Restaurant and Catering Services, Artvin Vocational School, Artvin Çoruh University, Artvin, Türkiye

Department of Forest Engineering, Faculty of Forestry, Karabük University, Karabük, Türkiye

Faculty of Science, Department of Chemistry, Karadeniz Technical University, Trabzon, Türkiye

Department of Biochemistry, Faculty of Pharmacy, Karadeniz Technical University, Trabzon, Türkiye

Nakhchivan State University, Nakhchivan, Azerbaijan

Karadeniz Technical University

Email: skolayli61@yahoo.com

Contents:

1. Supplementary Graps…………………………………………………………………..2

**1.1 Supplementary Figure S1(S1).** Calibration curve of gallic acid used for the determination of total phenolic content (TPC) by the Folin–Ciocalteu method…….2

**1.2 Supplementary Figure S2(S2)**. Calibration curve of quercetin used for the determination of total flavonoid content (TFC) by the aluminum chloride colorimetric method………………………………………………………………………………………...3

**1.3 Supplementary Figure S3(S3).** Calibration curve of Trolox was used for the determination of antioxidant capacity for FRAP assays. …………………………………4

**1.4 Supplementary Figure S4(S4).** Calibration curve of *M. elata* used for the determination of DPPH radical scavenging activity………………………………..……...5

**1.5 Supplementary Figure S5(S5).** Calibration curve of *M. esculenta* used for the determination of DPPH radical scavenging activity………………………………..……...6

**1.6 Supplementary Figure S6(S6).** HPLC chromatogram of *M. elata* extract showing identified phenolic compounds…………………………………………………...7

**1.7 Supplementary Figure S7(S7).** HPLC chromatogram of *M. esculenta* extract showing identified phenolic compounds…………………………………………………...8

Supplementary Graps

**Supplementary Figure S1**. Calibration curve of gallic acid used for the determination of total phenolic content (TPC).

**Supplementary Figure S2.** Calibration curve of quercetin used for the determination of total flavonoid content (TFC) by the aluminum chloride colorimetric method.

**Supplementary Figure S3.** Calibration curve of Trolox used for the determination of antioxidant capacity for FRAP assays.

**Supplementary Figure S4.** Calibration curve of *M. elata* used for the determination of DPPH radical scavenging activity

**Supplementary Figure S5.** Calibration curve of *M. esculenta* used for the determination of DPPH radical scavenging activity


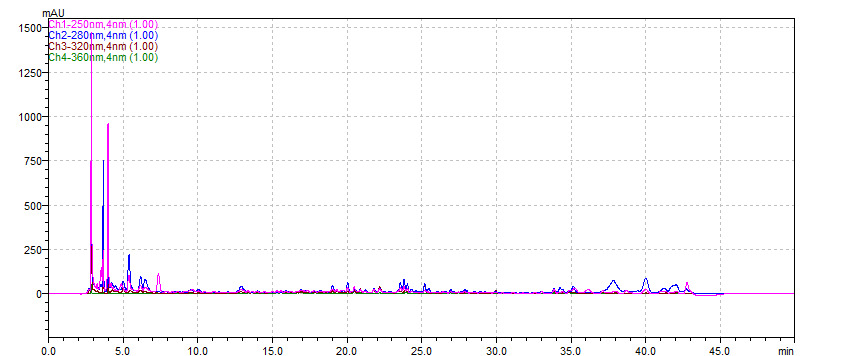


**Supplementary Figure S6.** HPLC chromatogram of *M. elata* extract showing identified phenolic compounds.


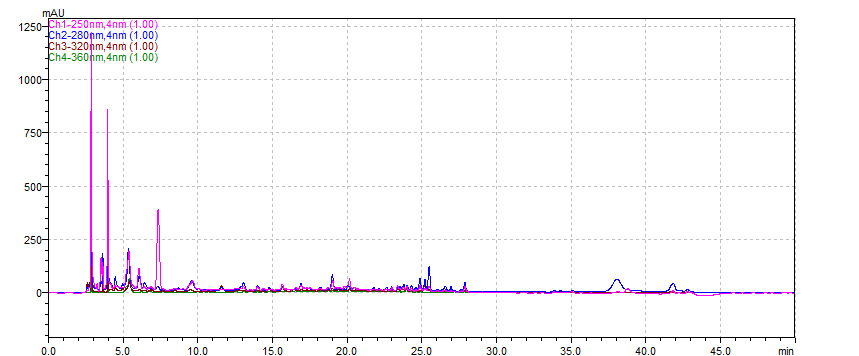


**Supplementary Figure S7.** HPLC chromatogram of *M. esculenta* extract showing identified phenolic compounds
